# Development of a miniaturized 3D organoid culture platform for ultra-high-throughput screening

**DOI:** 10.1093/jmcb/mjaa036

**Published:** 2020-07-17

**Authors:** Yuhong Du, Xingnan Li, Qiankun Niu, Xiulei Mo, Min Qui, Tingxuan Ma, Calvin J Kuo, Haian Fu

**Affiliations:** 1 Department of Pharmacology and Chemical Biology and Emory Chemical Biology Discovery Center, Emory University School of Medicine, Atlanta, GA, USA; 2 Department of Medicine, Division of Hematology, Stanford University School of Medicine, Stanford, CA, USA

**Keywords:** human colon organoids, KRAS^G12D^, 3D culture, 384-well plate, high-throughput screening (HTS), 1536-well plate, ultra-HTS (uHTS)

## Abstract

The recent advent of robust methods to grow human tissues as 3D organoids allows us to recapitulate the 3D architecture of tumors in an *in vitro* setting and offers a new orthogonal approach for drug discovery. However, organoid culturing with extracellular matrix to support 3D architecture has been challenging for high-throughput screening (HTS)-based drug discovery due to technical difficulties. Using genetically engineered human colon organoids as a model system, here we report our effort to miniaturize such 3D organoid culture with extracellular matrix support in high-density plates to enable HTS. We first established organoid culturing in a 384-well plate format and validated its application in a cell viability HTS assay by screening a 2036-compound library. We further miniaturized the 3D organoid culturing in a 1536-well ultra-HTS format and demonstrated its robust performance for large-scale primary compound screening. Our miniaturized organoid culturing method may be adapted to other types of organoids. By leveraging the power of 3D organoid culture in a high-density plate format, we provide a physiologically relevant screening platform to model tumors to accelerate organoid-based research and drug discovery.

## Introduction

High-throughput screening (HTS) is the process in which large compound libraries, ranging from thousands to millions of compounds, are tested for the activity against biological or disease-related targets. HTS has been widely used for early drug discovery in both industry and more recently in academia. It requires the use of automation, miniaturized assays, and large-scale data analysis (Broach and Thorner, 1996; Fox et al., 1999; Carnero, 2006; Inglese et al., 2007). Due to technical simplicity and ease of handling, traditionally cell growth and proliferation assays in 2D adherent cell monolayers on plastic surfaces have been widely used in the HTS field for the discovery of new cancer drugs (Breslin and O'Driscoll, 2013). However, transformed human cell lines cultured in 2D have complex mutational background and heterogeneity as a result of long-term passaging. Cell culturing in 2D does not account for the extracellular microenvironment, cell–cell interactions, and cell–matrix interactions normally found in tissues (Skardal et al., 2016). In addition, 2D culturing does not capture the disease heterogeneity observed *in vivo*. Consequently, the value of traditional 2D cell culturing in predicting clinical response has been challenged. Other cancer models, such as *in vivo* mouse models or the most advanced patient-derived xenograft mouse models, utilize mice to allow drug sensitivity testing for human patient tumors *in vivo*. However, these models are low throughput, restricted by cost and animal usage, and the tumors are grown in a mouse cancer context. A new technology platform that recapitulates *in vivo* properties of human tumors while allowing HTS operations for therapeutic discovery is much needed for accelerated discovery of novel agents in a disease-relevant environment. Emerging 3D organoid cancer models may address some of the deficiencies that exist with established human cancer cell lines and the animal models. Organoids, which recapitulate 3D structure and multilineage differentiation of diverse human tissues or tumors, represent a potentially powerful tool for HTS (Huch et al., 2013; Katano et al., 2013; Kim et al., 2019). Compared to the traditional 2D culture using transformed cell lines, primary organoids have the advantage of retaining the characteristics of the cancer cells from the original patient’s tissue, including their differentiated morphology and response to drugs (Clevers, 2016; Weeber et al., 2017; Abbasi, 2018; Sachs et al., 2018; Vlachogiannis et al., 2018). Therefore, organoids provide a new physiologically relevant disease model for drug discovery.

The use of organoids for HTS, however, is hampered by a number of technical challenges. It can be difficult to grow organoids to sufficient scale for conventional HTS formats. The requirement of extracellular matrix (ECM) to support the 3D architecture of organoids renders culturing in mini-wells of a high-density microplate for HTS, such as 384-well or 1536-well plates, highly challenging. Furthermore, the cost for organoid culturing reagents and ECM is quite high. Thus, a miniaturized format in high-density plates for streamlined and reproducible culturing of organoids will be essential for large-scale HTS. Most drug response testing using 3D organoids in 3D matrices is currently performed in lower throughput 96-well or larger well plates, mostly in a manual fashion, with limited number of testing compounds (Vlachogiannis et al., 2018; Li et al., 2019; Saito et al., 2019). Recent efforts have demonstrated the feasibility of using 384-well plate formats with 3D organoid cultures in Cultrex^®^ basement membrane extract (BME) gel (van de Wetering et al., 2015; Boehnke et al., 2016; Verissimo et al., 2016) or in Matrigel (Boehnke et al., 2016). Even so, 3D organoids have not been routinely applied to large-scale compound screening, e.g. >2000 compounds. With our past experience in assay development and HTS (Johns et al., 2014), through extensive optimization, we have achieved the miniaturization of 3D organoid culturing with BME support in both 384-well plates for HTS and 1536-well plates for ultra-HTS (uHTS). Using genetically engineered human colon KRAS^G12D^ organoids as a model system, we demonstrated the feasibility of our miniaturized 3D organoid culture platform for large-scale compound screening in a 1536-well uHTS format.

## Results

### Cryo-preserved frozen organoids for expansion

**Figure 1:**
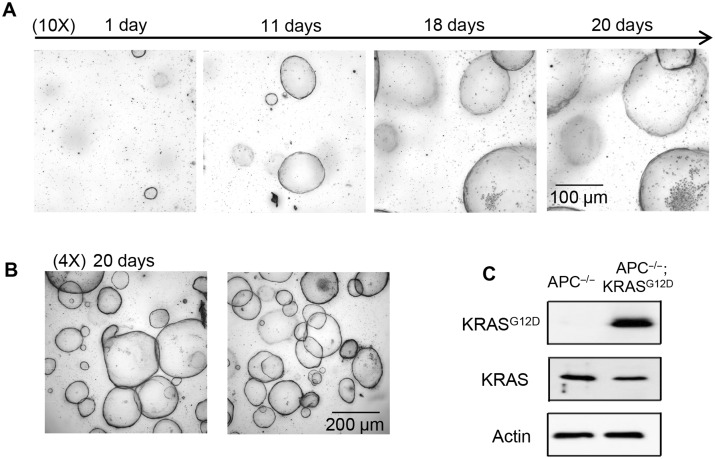
Morphological evaluation and biochemical confirmation of human colon organoids from cryo-preserved frozen samples. (**A**) Recovered human colon *APC^−/−^*;*KRAS^G12D^* organoids from frozen stocks. Frozen vials of organoids were thawed at 37°C, re-suspended in BME gel, and plated into a 24-well plate as a 50-µl droplet/well. The medium was refreshed every 3‒4 days. The growth of organoids was monitored with the ImageXpress^micro^ automated-imaging system (10× objective). (**B**) Robust growth of human colon *APC^−/−^*;*KRAS^G12D^* organoids (4× objective). Representative images from two different wells of a 24-well plate are shown. All the images shown are merged images of Z-stack using ImageXpress software. (**C**) The KRAS^G12D^ protein expression of human colon *APC^−/−^*;*KRAS^G12D^* organoids was confirmed in comparison to that of *APC^−/−^*;*KRAS^WT^* organoids. Organoids were collected for western blotting analysis with anti-KRAS^G12D^-specific antibody.

For large-scale screening, it is essential to generate organoids with sufficient numbers and consistent properties. One way to achieve this goal is to generate and cryo-preserve batches of organoids that could be recovered for specific applications. To determine whether organoids can be stored and recovered from frozen stocks in liquid nitrogen tanks as that for cell lines, we tested the recovery and growth of cryo-preserved organoids. Frozen oncogene-transformed human colon *KRAS^G12D^* organoids were established by CRISPR knock-in of the mutant *KRAS^G12D^* allele into human colon *APC^−/−^* organoids as will be described elsewhere. These human colon *APC^−/−^*;*KRAS^G12D^* organoids were shipped frozen and recovered in BME gel as 50 µl droplets in 24-well plates. Indeed, the recovered human colon *KRAS^G12D^* organoids from frozen vials exhibited progressive and robust growth as monitored using white field images over the course of 20 days (Figure 1A and B). The genotype of the engineered *APC^−/−^*;*KRAS^G12D^* organoids with the *KRAS^G12D^* mutation was verified using *KRAS^G12D^*-specific antibodies, showing the expression of *KRAS^G12D^* protein by western blotting analysis of cell lysates (Figure 1C). These data demonstrate that organoids can be cryo-preserved for long-term storage and re-grown upon cryo-recovery for expansion. These data support previous studies on cryopreservation of organoids for biobanking (van de Wetering et al., 2015; Sachs et al., 2018; Yao et al., 2020).

### Miniaturization of 3D organoid cultures in a 384-well plate format

**Figure 2:**
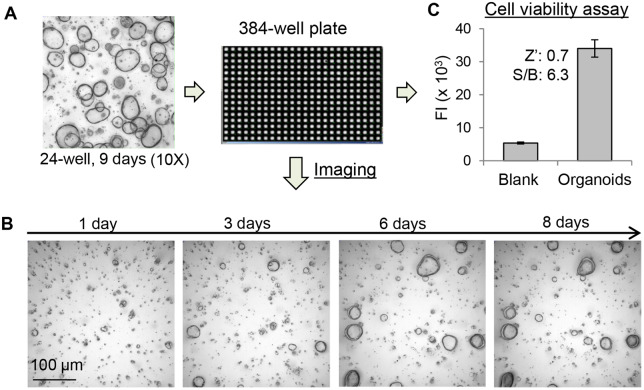
Culturing of human colon organoids in a 384-well plate format. (**A**) Scheme for miniaturizing organoid culture in a 384-well plate. Organoids growing in BME gel in the 24-well plate were collected and re-suspended in ice-cold BME gel. The organoid cells/BME gel mixture (8 µl/well) was dispensed into a 384-well plate. After incubation for 30 min at 37°C, warm organoid culture medium (35 µl) was dispensed to each well. The plate was incubated for up to 8 days without replacing the medium. (**B**) The organoids growing in a 384-well plate was monitored using ImageXpress^micro^ and showed progressing growth over the time. (**C**) The growth of organoids in 384-well plate was quantified by CellTiter Blue cell viability assay and showed robust FI signals for HTS with Zʹ of 0.7 and S/B ratio of 6.3.

To explore the feasibility of culturing organoids in a miniaturized plate format for HTS, we first optimized the growth conditions of organoids in a 384-well plate format by evaluating the ratio of the organoids/BME gel, dispensing methods and the volume of the matrix mixture, and the time course of organoid growth. As shown in Figure 2A, organoids growing in 24-well plates were collected, re-suspended in BME gel, and dispensed into wells of a 384-well plate. The growth of organoids over the incubation period was monitored by white light images using ImageXpress^micro^ automated imaging system. In Figure 2B, the organoids cultured in a 384-well plate showed progressive growth over time. After 3 days of culturing, the size of organoids became significantly larger compared with that of  the 1-day culture. Unlike the growth in a 24-well plate where organoids continued their growth over 20 days with repeated medium changes (Figure 1A and B), the growth of organoids in a 384-well plate reached a steady state around Day 7. Therefore, we selected Day 7 culture as the endpoint for compound testing in a 384-well plate.

To quantify the growth of organoids, the CellTiter Blue cell viability assay was used to monitor the metabolic activity of cells in the organoids. CellTiter Blue reagent was dispensed to 384-well plates and the fluorescence intensity (FI) was measured. The FI is correlated with numbers of viable cells in the well. As shown in Figure 2C, wells with organoids generated significant FI signal compared with that of blank control wells with medium only. For HTS applications, evaluation of well-to-well variations and the robustness of the assay are essential. Therefore, the Zʹ factor and the signal-to-background (S/B) ratio parameters were calculated. The Zʹ factor is used to evaluate performance uniformity of the assay for HTS without testing compounds. Zʹ >0.5 and <1 indicates the assay is robust with minimal variation for screening (Zhang et al., 1999). Indeed, the Zʹ of the organoid viability assay in a 384-well plate was 0.7 and S/B was 6.3, exhibiting robust performance suitable for HTS in this format.

### Utilization of the 3D organoid culture in a 384-well format for HTS

To demonstrate the utility of the 384-well cultured organoids for HTS, we carried out a cell viability screening with the Emory Enriched Bioactive Library (EEBL) (Mo et al., 2019). This library includes 2036 FDA-approved and bioactive compounds and was screened against the above colon *APC^−/−^*;*KRAS^G12D^* organoids. The organoids were seeded into 384-well plates and incubated for 4 days to allow organoid growth before testing compounds were added. Upon incubation for 3 days, CellTiter Blue reagent was used to evaluate cell viability.

**Figure 3:**
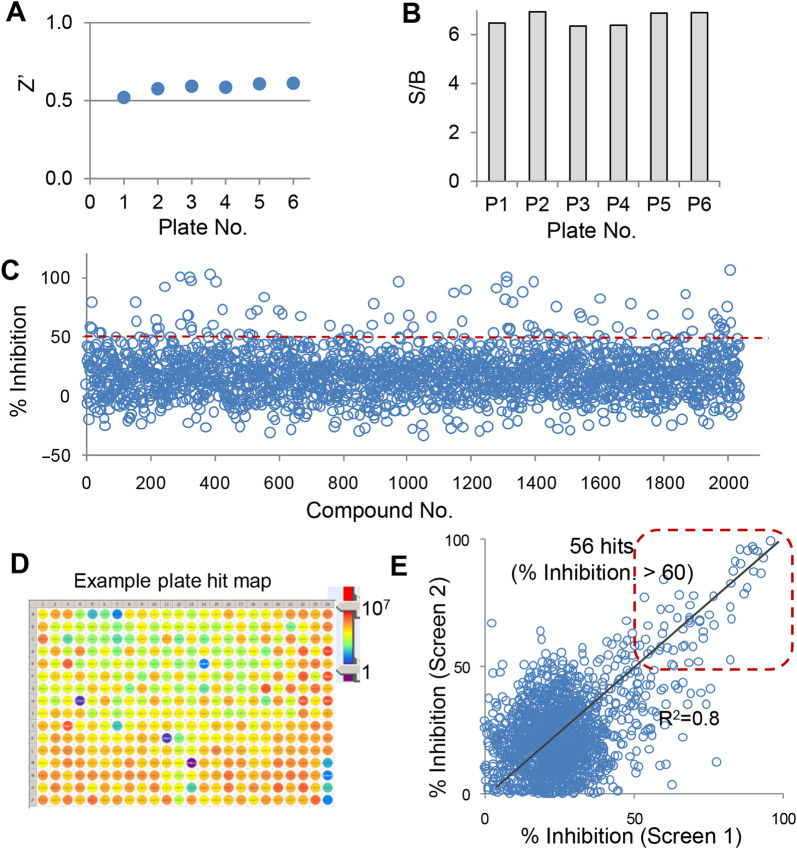
Screening of the EEBL library with the human colon organoids in a 384-well plate format. Human colon *APC^−/−^*;*KRAS^G12D^* organoids cultured in 384-well plates were treated with library compounds (0.1 µl, final at 4.6 µM). The plates were incubated for 3 days and subjected to CellTiter Blue cell viability assay. The effect of compounds on the growth of KRAS^G12D^ organoids was calculated as % Inhibition based on each plate and analyzed using Cambridge BioAssay software. (**A** and **B**) Performance evaluation with Zʹ factors (**A**) and S/B (**B**) across six 384-well screening plates. (**C**) Scatter plot of compound effects (% Inhibition) from the HTS. (**D**) An example plate hit map. (**E**) Correlation between two independent screens.

We first evaluated the performance of the screening using the set of control wells without organoids in each plate. Each screening plate included one column (16 wells) of DMSO control for the maximum signal of organoids and one column (16 wells) of blank control for the minimal signal. The Zʹ and S/B were calculated for six 384-well screening plates. The organoids cultured in 384-well plates gave rise to significant FI signal with the CellTiter Blue viability assay. The Zʹ factors were all >0.5 (Figure 3A) and S/B were all >6 (Figure 3B), indicating a robust and high-quality HTS assay for the screening.

The effect of library compounds on organoid growth was examined and expressed as % Inhibition based on the controls of each plate. The results of the screening with the 2036-compound library are shown as the scatter plot in Figure 3C. Fifty-six primary hits were obtained based on the hit cutoff at % Inhibition >60. An example hit map from one screening plate is shown (Figure 3D). To evaluate the day-to-day and batch-to-batch reproducibility, the screen was repeated on a different day with a different passage of organoids. As shown in Figure 3E, we observed tight correlation between two independent screens, demonstrating the consistency and reproducibility of the organoid HTS viability assay.

**Figure 4:**
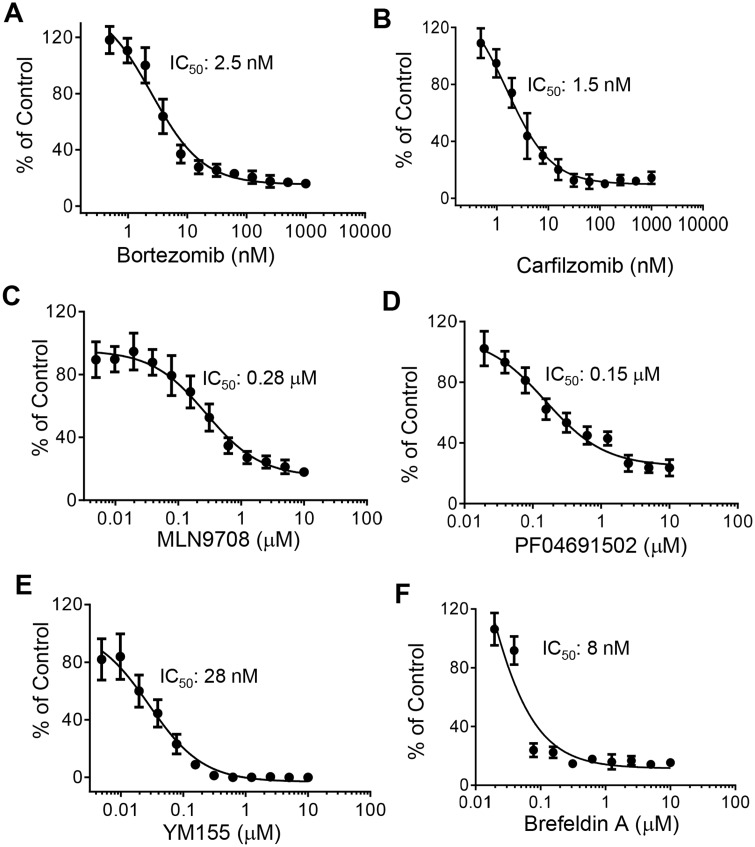
Dose‒response curves of selected top hits. (**A**‒**C**) Three proteasome inhibitors, bortezomib (**A**), carfilzomib (**B**), and MLN9708 (**C**), showed potent inhibitory effect on the growth of organoids. (**D**‒**F**) The PI3K/mTOR inhibitor PF04691502 (**D**), the survivin inhibitor YM155 (**E**), and the protein transporter inhibitor brefeldin A (**F**) inhibited organoid growth in a dose-dependent manner. Tested hit compounds were re-purchased for the dose‒response study. The effect of compounds on organoid growth was expressed as % of Control. Data shown are mean from triplicate samples with SD and analyzed using GraphPad Prism software.

To confirm the results from the primary screening, top hits were cherry-picked from the library stock and tested in a dose‒response confirmatory screen with triplicates per sample. Forty-three compounds passed the dose‒response validation. Confirmed hits that showed IC_50_ < 3 µM were re-purchased and re-tested in a dose‒response format with a different batch of the *APC^−/−^*;*KRAS^G12D^* organoids. A number of positive hits showed consistent inhibitory effects. Two proteasome inhibitors, bortezomib (Figure 4A) and carfilzomib (Figure 4B), exhibited dose-dependent inhibition of organoid growth with IC_50_ around 2.5 and 1.5 nM, respectively. MLN 9708, another proteasome inhibitor, also exhibited a similar effect with IC_50_ of 280 nM. These data suggest that the KRAS^G12D^-driven tumors may depend on proteasome function for their survival. Additional compounds, PF04691502, a PI3K/mTOR inhibitor (Figure 4D), YM155, a survivin inhibitor (Figure 4E), and brefeldin A, a protein transporter inhibitor (Figure 4F) showed significant inhibitory effect on organoid growth. The IC_50_ for PF04691502, YM155, and brefeldin A were 150, 28, and 8 nM, respectively. These results suggest that the 384-well viability assay format for the 3D organoids offers robust performance and is able to reveal differential compound effects for hit identification.

### Orthogonal imaging-based assay for hit confirmation

**Figure 5:**
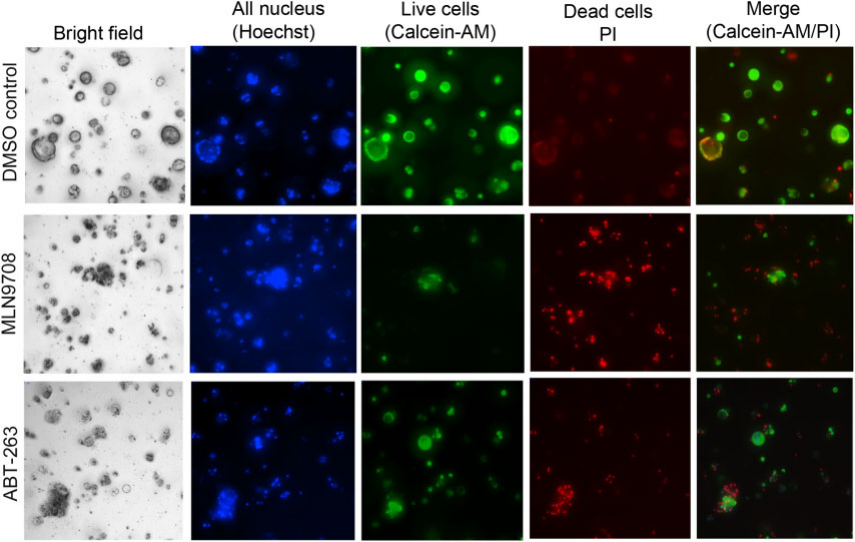
Imaging analysis of cell fate of organoids in a 384-well plate format. Organoids growing in a 384-well plate (4 days) were treated with DMSO, MLN9708, or ABT-263 (5 µM) for 3 days. A combination of dyes diluted in PBS containing Hoechst 33342, Calcein-AM, and PI was added and incubated for 30 min. The bright field and fluorescence images were captured with the ImageXpress^micro^ automated imaging system using Z-stack. The representative merged images from Z-stack for DMSO control, MLN9708, and ABT-263 treatment are shown.

To further confirm the compound effects with an orthogonal approach, we developed an imaging-based assay to monitor the phenotypic change of live and dead cells of organoids in a 384-well plate format. In this assay, we used a three-dye system with Hoechst 33342 for cell tracking, Calcein-AM for live cells, and propidium iodide (PI) for dead cells through staining nucleus, live, and dead cells, respectively (Jiang et al., 2016; Chan et al., 2017). As shown in Figure 5, control organoids with DMSO treatment exhibited a donut-shaped morphology under bright field, with an integrated ‘ring’ structure at outer edge and shallow in the center. Live-cell staining with Calcein-AM showed a similar pattern with bright green fluorescence at the outer edge. Only weak PI staining for dead cell was observed (upper panel). After the organoids were treated with hit compounds, such as MLN9708 as shown in the middle panel and ABT-263 in the bottom panel, the morphology of organoids changed compared with that of the DMSO control (Figure 5). The integrity of organoids’ ‘ring’ structures disappeared and the organoid shapes became disorganized with aggregated dark spots under white field images. Furthermore, the drug-treated organoids exhibited weak green fluorescence from Calcein-AM live-cell staining and strong PI staining signal, indicating reduced viable cells and increased number of dead cells. These imaging results are consistent with cell viability assay data, providing a complementary approach to further validate the effect of compounds on the growth properties of organoids.

Together, these results from the pilot screening and the dose‒response confirmatory assay suggest that the 3D organoid viability assay in a 384-well format allows the identification of hit compounds. We identified and validated a panel of compounds that have shown high potency on the growth inhibition of human colon organoids with *KRAS^G12D^* mutations. These results support the scale-up of compound screening for developing KRAS^G12D^-targeted therapeutics.

### Miniaturization of the 3D organoid culture system in a 1536-well plate format for uHTS

**Figure 6:**
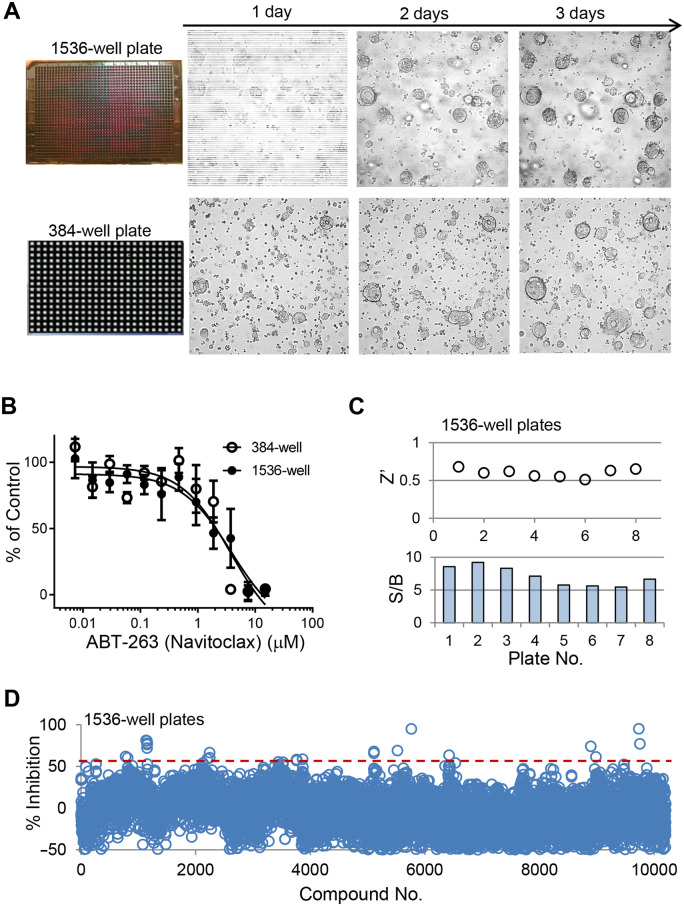
Miniaturized 3D organoid assays in a 1536-well plate format for uHTS. (**A**) Growth of human colon *APC^−/−^*;*KRAS^G12D^* organoids in 1536-well vs. 384-well plates was compared. (**B**) The effect of compounds, ABT-263 as an example, exhibited very similar inhibitory effect on the growth of *APC^−/−^*;*KRAS^G12D^* organoids between 384-well and 1536-well plate formats. (**C**) Performance evaluation for the uHTS viability assay with organoids showing Zʹ (>0.5) and S/B (>5) scores across the screening plates. (**D**) Scatter plot of primary screening of a 10240-diversity compound library in a 1536-well uHTS format. Screening data were analyzed using Cambridge BioAssay software. The effect of compounds on the growth of *APC^−/−^*;*KRAS^G12D^* organoids was calculated as % Inhibition per plate.

To further scale up the organoid screening, additional miniaturization of the culturing system is needed. One of the major bottlenecks for large-scale 3D organoid screening is the expansion and growth of a sufficient number of organoids. Reducing the volume needed with enhanced detection sensitivity might help address this challenge. To reduce the growth culture volume and enable efficient large-scale screening, we examined the possibility of further miniaturizing the 3D organoid culturing system to allow the growth of 3D *APC^−/−^*; *KRAS^G12D^*organoids in a 1536-well plate format. Optimization of culturing conditions and streamlined operational procedures enabled fast processing of organoid seeding and efficient cooling for 3D matrix dispensing with BME gel. The growth of organoids in a 1536-well format was monitored by bright field imaging. As shown in Figure 6A, the 1536-well plate organoids showed progressive growth over time (top panel), with similar growth pattern to the 384-well plate (bottom panel). To test the feasibility of using the 3D uHTS organoids system for compound evaluation, we compared the effect of a selected hit compound from the 384-well pilot screening, ABT-263, on the growth of organoids between 1536-well and 384-well plate formats. As shown in Figure 6B, the dose‒response curves of the compound tested in a 1536-well plate and that tested in the 384-well format were almost identical, showing similar performance of the growth inhibitory effect of the test compound in both HTS and uHTS format.

To advance the 1536-well organoid uHTS viability assay for screening as a proof of concept, we carried out a primary screen with a diverse chemical library of 10240 compounds in 1536-well plates. To assess the quality of the screening in such an uHTS format, we evaluated the well-to-well and plate-to-plate variations. As shown in Figure 6B, the Zʹ factor was >0.5 and S/B was >5 across the screening plates, demonstrating a robust and sensitive assay performance. The screening results were shown as a scatter plot in Figure 6C. From this uHTS, 18 primary hits were identified with hit cutoff at % Inhibition >60. These hits will be further characterized with additional assays in separate studies.

**Figure 7:**
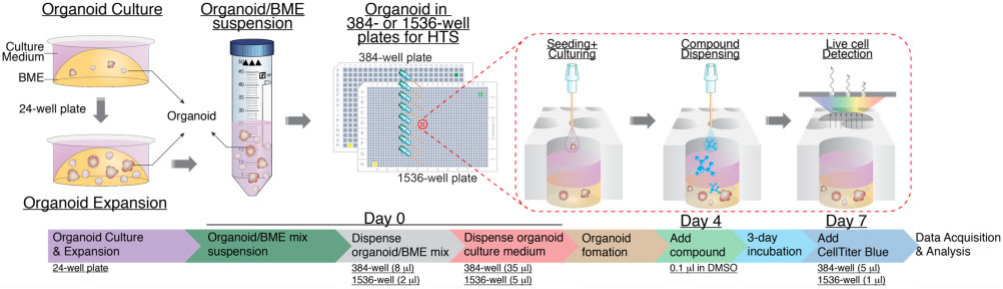
Work flow of 3D organoid culture for compound screening in 384-well HTS and 1536-well uHTS formats.

Altogether, these results demonstrate the feasibility of our optimized 3D 1536-well uHTS format for organoid viability screening. The expanding and utilization of our uHTS organoid platform will enable large-scale primary compound screening to accelerate 3D organoid-based drug discovery. In summary, we have established a cost-effective protocol for 3D organoid compound screening in both 384-well and 1536-well plate formats. The work flow we describe here, as summarized in Figure 7, could be adapted to suit various HTS automation systems.

## Discussion

Early drug discovery relies on the use of 2D cell-based assays to screen and determine the potency of active lead compounds. Due to its simplicity and easy handling, 2D cultures of established cell lines have been widely used for compound screening, drug sensitivity testing, and pharmacogenomics profiling. Such established cell lines can be maintained using standardized culturing methods. It is relatively straightforward to culture and prepare large quantities of cells for HTS and uHTS. However, 2D cultured cells often exhibit a flattened morphology and altered signaling networks compared to cancer cells in tumor tissue *in vivo* (Weaver et al., 2002). Thus, the response to drugs in cancer cells may not always accurately reflect their response *in vivo* (Weaver et al., 2002). Therefore, physiologically relevant models for screening are highly desirable in order to increase the returns of HTS-enabled drug discovery efforts.

Organoid-based technologies have been widespread in recent years due to their unique 3D structures that recapitulate organ structure, multilineage differentiation, and physiology. They represent near-physiological models for use in both basic and translational research (Drost and Clevers, 2018; Yang et al., 2018; Castellon, 2019; Kim et al., 2019). In addition, organoids can be expanded, cryo-preserved in biobanks, and manipulated by genetic modification using modern techniques similar to those established for traditional 2D culture of transformed cell lines. Importantly, it has been shown that patient-derived organoids recapitulate histological and genetic features of original tumors and the responses of patients to drugs in the clinic (Sachs et al., 2018; Vlachogiannis et al., 2018). 3D organoid-based screening platforms as a new generation of physiologically relevant models are expected to bridge the gap between traditional 2D cultures and *in vivo* mouse models to facilitate drug discovery and personalized medicine. Despite numerous potential advantages, organoids have been vastly underutilized for drug discovery due to many challenges to implement 3D organoids for HTS. Robust execution of HTS requires a sufficient number of cells/organoids. Culturing and propagating 3D organoids is not only dependent on the heterogeneous nature of organoid growth, which is beyond the scope of this study but also requires special technical skills. While optimizing conditions to enable scale-up organoid culture will be valuable, miniaturization of the 3D organoid culturing system to a 1536-well format will not only enable screening a large number of chemicals with a limited number of organoids, but also will drastically reduce the cost of large-scale screening.

Even though 3D spheroid-based viability assay has been successfully miniaturized for drug screening, including in 1536-well plate format (Madoux et al., 2017; Griner et al., 2018; Hou et al., 2018), culturing of such spheroids typically does not require the use of ECM to support their 3D architecture. They are freely floating cell aggregates from a single cell type or from a mixture of cells that are often cultured in ultra-low attachment plates to promote cell self-aggregation into sphere-shaped 3D structures. Unlike traditional 2D cell culture or the 3D spheroid culture, 3D organoid culture requires a semi-solid form of ECM to support organoid growth within a 3D architecture. ECM is vital to support epithelial stem cell proliferation, differentiation, and propagation and to maintain their form as an organoid. Matric components include laminin, collagen IV, entactin, and heparan sulfate proteoglycans that may induce ECM signaling suitable for organoid formation. Currently, the most commonly used ECM for organoid culture is a soluble form of gelatinous protein mixture extracted from mouse Engelbreth-Holm-Swarm (EHS) tumor tissue, which provides a natural ECM hydrogel that polymerizes at 37°C to form a reconstituted basement membrane. Two commercial products, Cultrex^®^ BME by Trevigen and Matrigel by Corning, have both been successfully used to support 3D organoid culture. However, handling ECM for automated HTS operations presents challenges, as it is supplied as frozen stock stored at −80°C, maintains soluble form at cold temperatures (4°C), and solidifies easily while processing at room temperature. HTS requires robust assay platforms amendable to automation with minimal day-to-day or batch-to-batch variations. In an HTS setting, a liquid dispenser is generally used to automatically dispense cells or reagents into 384/1536-well plates. Most of the dispensers, e.g. Multidrop Combi dispenser, use specialized dispensing tubing or cassettes. One end of the tubing is inserted into a conical tube containing cells or reagents and the other end with eight-channel pipetting tips that is attached into instrument in alignment with the Society for Biomolecular Screening (SBS) standard plate dimensions for precisely dispensing into 96/384/1536-well plates. Due to the high viscosity of the soluble form of BME gel and its polymerization feature upon exposure to warm environment, such as room temperature, it is easy to block the dispensing tubing and tips, especially for low-volume dispensing tubing (<10 µl). Dead volume is also required to fill up the tubing before dispensing into microplates. Therefore, extra volume of at least 2 ml is normally required. The cost of BME gel or Matrigel and organoid culture medium is relatively high. Therefore, in order to enable 3D organoid screening using Matrigel or BME as supporting matrix, miniaturization to achieve reduced volume and robust readouts is essential, yet challenging. Currently, only small scales, e.g. <100 compounds, have been tested as proof-of-concept to exam the response of 3D organoids in ECM to drugs (Gao et al., 2014; van de Wetering et al., 2015; Schutte et al., 2017; Sachs et al., 2018; Li et al., 2019). The use of 3D organoids in ECM for large-scale compound screening has not been reported in the HTS field so far. Our work reported here represents a major advancement in culturing the organoids in a 3D matrix in 384-well and 1536-well plate format that enables uHTS campaign.

We utilized the genetically engineered organoids to model colorectal cancer (CRC) with an oncogenic KRAS mutation as a model system for HTS and uHTS platform development. KRAS mutations are amongst the most pervasive genetic alterations in human cancer (Wood et al., 2007). CRC is one of the most common cancers worldwide (Ferlay et al., 2015). However, direct therapeutic targeting of constitutively active mutant KRAS has proven challenging despite considerable effort. Attempts to develop targeted therapies for this genomic subset of patients have been largely unsuccessful, although therapeutic targeting of *KRAS^G12C^* tumors has now advanced to phase II clinical trials, offering much hope for patients (Canon et al., 2019). Thus, establishing a screening platform to target cancers with KRAS mutations is expected to have significant implications.

The use of genetically transformed mutant KRAS organoids derived from primary wild-type organoids has numerous advantages over transformed cell lines. The primary wild-type organoids maintain native differentiation and 3D tissue architecture and allow mutant KRAS-targeting screening to be performed in well-defined human genetic backgrounds devoid of confounding mutations, which commonly occur in long-term passaging of 2D cancer cell lines. Previously, we used genetically engineered mouse colon KRAS^G12D^ organoids derived from WT organoids of normal mouse colon with KRAS^G12D^ oncogene transformation as a model system (Ootani et al., 2009; Li et al., 2014). Here we used CRISPR to knock-in KRAS^G12D^ into human colon *APC^−/−^* organoids, which was used as starting material to first develop and miniaturize a 3D organoid growth assay in a 384-well format for HTS using automation. For our organoid cultures, we used Cultrex^®^ reduced growth factor BME as supporting ECM, which is processed to reduce matrix-associated growth factors and provides a defined model system for organoid culture. Consistent with our selection, BME has been used successfully for supporting many tissues or tumor-derived organoid culture as a 3D scaffold (Crespo et al., 2017; Sachs et al., 2018; Suzuki et al., 2018).

Our application of the HTS viability assay with KRAS^G12D^-carrying 3D organoids was validated in a pilot screen of 2036 FDA-approved and bioactive compounds. Dose‒response confirmatory screening provided further evidence for a panel of compounds that inhibit the KRAS^G12D^ organoid growth, raising the possibility of KRAS^G12D^-dependent targets or pathways. Our results support the recent clinical trial of bortezomib in non-small cell lung cancer with two exceptional responders, both of whom had KRAS^G12D^ mutant tumors (Drilon et al., 2019). We also confirmed two proteasome inhibitors, carfilzomib and MLN9708, which potently inhibit the growth of KRAS^G12D^ organoids. These results are consistent with previous studies that KRAS-mutant tumor cells have increased proteasomal activity and are vulnerable to proteasome inhibition (Luo et al., 2009; Steckel et al., 2012). In addition to proteasome inhibitors, we have confirmed that two additional compounds, the PI3K/mTOR inhibitor PF04691502 and the survivin inhibitor YM155, attenuate the growth of KRAS^G12D^ organoids. Previous studies have indicated the role of PI3K signaling activation in initiating KRAS^G12D^-driven tumorigenesis (Castellano et al., 2013; Green et al., 2015). High expression levels of survivin, encoded by the *BIRC5* gene, in KRAS-mutant lung adenocarcinomas are significantly associated with poorer patient outcomes (Sumi et al., 2018). These results suggest that the KRAS^G12D^ organoid-based HTS viability assay platform may offer an effective approach for the discovery of mutant KRAS-targeted therapeutics. Interestingly, brefeldin A, a fungal product, efficiently inhibits the growth of KRAS^G12D^ organoids. Brefeldin A is a specific inhibitor of protein trafficking, which blocks the protein transport from the endoplasmic reticulum (ER) to the Golgi complex, leading to ER stress and Golgi stress, and the induction of apoptosis (Zhu et al., 2000). How brefeldin A functions to affect KRAS signaling remains to be established.

To further increase the throughput after optimization, we have successfully miniaturized our 3D organoid culture into a 1536-well uHTS format. The robustness and assay performance of the miniaturized uHTS viability assay for screening were assessed by a 10240 compound screen. The reduced volume of 2 µl of cells mixed with BME per well in a 1536-well plate not only significantly decreased the number of organoids needed for screening but also reduced the cost for BME or other ECM matrix needed. Reduced volume also allows cost-saving for expensive organoid culture medium. This miniaturized organoid culture system with the support of the 3D matrix in a 1536-well format is readily applicable for scaling up for primary compound screening.

Moreover, our established protocols for miniaturized 3D organoid culture in 384-well and 1536-well formats could be adapted for other types of organoids. Even though BME gel was used in this study, we do not expect technical challenges with using other types of ECM, such as Matrigel. Combining the power of primary organoids with a defined genetic background with advanced HTS technologies, our miniaturized 3D organoid culture platform provides an enabling technology to accelerate basic and translational research using physiologically relevant disease models.

## Materials and methods

### Materials

BME gel (Cultrex^®^ reduced growth factor basement membrane extract, type 2 (RGF BME-2), 3533-005-02) was purchased from Trevagen. Advanced DMEM/F12 medium, N2 supplement, B-27 supplement, HEPES, GlutaMAX-I, Penicillin and streptomycin, G-418, and hygromycin B were obtained from Invitrogen. [Leu15]-gastrin 1 human, nicotinamide, N-acetylcysteine, human insulin, human transferring, and fetal bovine serum (FBS) were obtained from Sigma-Aldrich. A83-01 was acquired from R&D Systems Inc. SB202190, Y-27632, and CHIR99021 were purchased from Cayman Chemical. FGF10 and EGF were purchased from PeproTech. TrypLE express and phosphate-buffered saline (PBS) were obtained from Gibco-Thermo Fisher Scientific Inc.

### WRN (Wnt3A, RSPO1, Noggin)-conditioned medium

WRN-conditioned medium was prepared using L-WRN cells that were purchased from ATCC (CRL-3276). The L-WRN cells were derived by transfecting L-Wnt3A cells (ATCC CRL-2647) with an R-spondin 3 and Noggin co-expressing vector. Briefly, the cells purchased from ATCC were recovered and cultured with Dulbecco’s modified Eagle’s medium (ATCC, 30-2002) supplemented with 10% FBS, 0.5 mg/ml G-418, and 0.5 mg/ml hygromycin B. Once the cells reached confluence, they were sequentially split and cultured in T-150 flasks (25 ml cell suspension). Upon reaching confluence, the medium was collected by centrifugation (2000× *g*, 5 min) and stored at 4°C. This is the batch 1 of conditioned medium. The remaining cells in the T-150 flasks were continuously cultured by adding 25 ml of medium for 24 h to repeat the process of collecting additional batches of conditioned medium, which are combined and stored in aliquots at −20°C before use.

### Medium for human colon organoid culturing and passaging

The complete organoid culture medium was prepared, which includes the following components: advanced DMEM/F-12 cell culture medium with 50% WRN-conditioned medium, HEPES (1 mM, pH 7.4), 1× glutamax, nicotinamine (10 mM), N-acetylcysteine (1 mM), 1× B27 supplement, 1× N2 supplement, [Leu15]-gastrin 1 human (10 nM), A83-01 (0.5 µM), SB202190 (10 µM), human transferrin (7.5 µg/ml), human recombinant EGF (50 ng/ml), recombinant human FGF (100 ng/ml), and normocin (100 µg/ml). Organoid starting/passing medium was prepared by adding Y-27632 (Rho kinase inhibitor; 10 µM) and CHIR 99021 (GSK-3 inhibitor; 2.5 µM) into complete organoid culture medium.

### Recovery of organoids from frozen stock

Human colon KRAS^G12D^ organoids were generated by engineering normal colon tissue organoids and characterized as previously described (Ootani et al., 2009). The frozen organoids were shipped and thawed quickly in a 37°C water bath and washed five times using PBS (500× *g*, 5 min). The cell pellets were then re-suspended in ice-cold BME gel and placed in the center of a 24-well plate (50 µl droplet/well). After incubating at 37°C till the BME gel was solidified, organoid passaging medium (0.5 ml) was added to the wells. The organoids were incubated in the cell culture incubator at 37°C with 5% CO_2_. The medium was replaced and refreshed with complete organoid culture medium every 3 days.

### Organoid culture expansion

The organoids growing in 24-well plates were collected and placed in a 15-ml conical tube. After washing with PBS once (1000 rpm, 5 min), TrypLE express was added and incubated in a 37°C water bath for 15 min to dissociate the cells. Trypsinization was stopped with FBS, and digested cell clusters were washed three times with PBS. During the PBS washing, the tube containing cells in PBS was mixed well by inverting upside down for >5 times to dissociate the cells further mechanically. The cell pellets were re-suspended in ice-cold BME gel at desired volume (normally 1:3 ratio splitting) and re-plated as a 50-µl droplet/well in a 24-well plate. The organoids were initially cultured with 0.5 ml/well passaging medium for 3 days after re-plating and then refreshed with 0.5 ml/well completed organoid culture medium every 3 days.

### Western blotting analysis

The KRAS^G12D^ expression of organoids was determined by western blotting analysis. After dissociating organoids from BME gel by TrypLE express and harvesting the cells, cells were washed with PBS buffer, then lysed in 0.5% Triton buffer (20 mM Tris, pH 8.0, 137 mM NaCl, 5% glycerol, 0.5% Triton X-100, Protease Inhibitor (Sigma, P8340)), and centrifuged for 10 min at 4 °C. Total protein was quantified with BCA assay (Thermo Fisher, 23227). Equal amounts of the protein were mixed with 2× Laemmli sample buffer (BioRad, 161-0737), incubated at 95°C for 5 min, and then subjected to sodium dodecyl sulfate-polyacrylamide gel electrophoresis (SDS‒PAGE). Western blotting was carried out following a standard protocol. The membrane was incubated with primary antibody in TBST buffer at 4°C overnight, followed by incubation with corresponding horseradish peroxidase-conjugated secondary antibody for 1 h at room temperature, and then visualized using the SuperSignal West Pico PLUS Chemiluminescent Substrate (Thermo Fisher, 34580). The following antibodies were used: anti-KRAS at 1:1000 (Proteintech, 12063-1-AP); anti-RAS^G12D^ at 1:1000 (Cell Signaling Technology, 14429); anti-β-actin at 1:5000 (Sigma, A5441); goat-anti-rabbit IgG at 1:5000 (Jackson ImmunoResearch, 111-035-003), and goat-anti-mouse IgG at 1:5000 (Jackson ImmunoResearch, 115-035-003).

### Miniaturization of organoid culture and viability assay in a 384-well HTS format

The organoids growing in 24-well plate were harvested as described above and re-suspended in ice-cold BME gel to form the cells/BME gel mixture. The cells/BME gel mixture (8 μl/well) was dispensed into 384-well plates using Multidrop Combi dispenser (Thermo Fisher Scientific). To avoid the BME gel solidified during dispensing, the dispensing cassette was pre-chilled at 4°C and the 50-ml conical tube containing cells/BME gel mixture was placed in ice during dispensing process. The plates were immediately centrifuged at low speed, e.g. 300× *g*, for 1 min to ensure the uniform distribution of the cells/BME gel in the wells across the entire plate. Centrifugation also helps to remove any bubbles during dispensing. After incubating the plates at cell culture incubator for 30 min, 35 µl of warm completed organoid culture medium was dispensed to each well. The plates were then sealed with gas-permeable plate sealer (Breathe-Easy Sealing Film, Diversified Biotech, BEM-1) and incubated in the cell incubator.

The viability of organoids in 384-well plate was determined by CellTiter Blue reagent (Promega). Briefly, at the end of the organoid culturing process, CellTiter Blue reagent (5 µl) was added to each well in 384-well plates. After incubation at 37°C for 4 h, the FI was measured using the PHERAstar FSX multi-label plate reader (BMG LABTECH) with excitation (Ex.) at 540/20 nm and emission (Em.) at 590/20 nm.

### HTS with the organoid viability assay in 384-well plates

The EEBL that contains a total of 2036 compounds was used for HTS. The collection includes FDA-approved and bioactive compounds with known activities, targeting >20 signaling pathway (Mo et al., 2019). Organoids were cultured in 384-well plates and incubated at 37°C as described above to allow organoid formation. Library compounds diluted in DMSO (0.1 µl) were added to each well using Pin-tool integrated with Beckman NX automated liquid handling system (Beckman Coulter, Danaher Corporation). To avoid the pin-tool inserted into cells/BME gel layer at the bottom of the well, the pin-tool dispense height was carefully optimized and set at the height that the bottom of pin-tool was above the cells/BME gel layer while still inside the medium layer for accurate compound transfer. The plates were centrifuged at 600× *g* for 5 min to ensure the uniform distribution of the compound into wells. The final compound concentration was 4.6 µM and the final DMSO was 0.2%. The plates were sealed with gas-permeable plate sealer. After incubation with compounds for 3 days, the CellTiter Blue reagent (5 µl/well) was dispensed to each well. The plates were incubated for 4 h and FI was measured using PHERAstar FSX.

### Organoid image capture in multi-well plates using the automated imaging system

The phase images of the organoids growing in 24-, 384-, or 1536-well plates were captured using ImageXpress^micro^ XLS (Molecule Devices) using 10× objective. Z-stack was applied with minimum projection to merge the Z-stack images using ImageXpress software.

### Multiplexed imaging of organoids in 384-well plates

Calcein-AM and PI (Invitrogen) were used to monitor the live and dead cells of organoids in a 384-well plate format. Hoechst 33342 (Invitrogen) was used for staining nucleus. For this assay, 5 µl of staining solution containing Calcein-AM (final at 4 µM), PI (final at 10 µg/ml), and Hoechst 33342 (final at 10 µg/ml) was added to the wells of a 384-well plate. After incubation for 30 min, the images were acquired using ImageXpress^micro^ with Z-stacks for each channel. The FITC filter set with Ex. 482/35 nm and Em. 536/40 nm was used for imaging Calcein-AM-stained live cells and the TXRED filter set with Ex. 562/40 nm and Em. 624/40 nm was used for imaging dead cells stained with PI.

### Miniaturization of organoid growth and viability assay in a 1536-well plate format for uHTS

Organoids growing in 24-well plates were harvested and mixed with ice-cold BME gel. The cells/BME gel mixture (2 µl/well, ∼400 cells) was dispensed into a black-wall clear-bottom 1536-well plate (Corning, 3893) using a Multidrop Combi dispenser. The dispensing cassette was pre-chilled at 4°C and the cells/BME gel mixture was placed in ice during dispensing. The plates were immediately centrifuged as described above. After incubating the plate for 30 min to allow BME gel solidified, 5 µl warm organoids growth medium was dispensed to each well. The plate was sealed with gas-permeable sealer and incubated in a cell culture incubator. For compound testing and library screening, testing compounds (0.1 µl) were added into each well using pin-tool as described above. The plates were centrifuged at 600× *g* for 5 min and sealed with the gas-permeable sealing membrane. After incubation with compounds for indicated days, the CellTiter Blue reagent (1 µl/well) was dispensed into wells. The plates were incubated for 6 h and FI was measured using PHERAstar FSX.

### Data analysis

Screening data were analyzed using the CambridgeSoft Bioassay software. The performance of the organoid viability assay in 384-well and 1536-well plates for HTS or uHTS was evaluated by Z’ factor and S/B ratio, which were calculated as the following equations:

$$Z\prime =1\;–\;\left({3\text{SD}}_{\text{DMSO}\;\text{control}}+{\;3\text{SD}}_{\text{blank}}\right)/\left({\text{FI}}_{\text{DMSO}\;\text{control}}–{\;\text{FI}}_{\text{blank}}\right)$$

and

$$S/B={\;\text{FI}}_{\text{DMSO}\;\text{control}}/{\text{FI}}_{\text{blank},}$$

where SD_DMSO control_ and SD_blank_ are the standard deviations and FI_DMSO control_ and FI_blank_ are the corresponding average FI signals for the wells with DMSO control and blank with medium only without cells, respectively. A Zʹ factor between 0.5 and 1.0 indicates that the assay is robust for HTS (Zhang et al., 1999).

The effect of compound on the growth of organoids was expressed as % of Control or % Inhibition based on per plate and calculated as the following equations:

$$\%\;\text{of}\;\text{Control}=\left({\text{FI}}_{\text{compound}}–{\;\text{FI}}_{\text{blank}}\right)/\left({\text{FI}}_{\text{DMSO}\;\text{control}}–{\;\text{FI}}_{\text{blank}}\right)\times 1\mathrm{00}$$

and

% Inhibition=100 – % of Control.

## References

[mjaa036-B1] Abbasi J. (2018). Patient-derived organoids predict cancer treatment response. JAMA 319, 1427. 10.1001/jama.2018.3760 29634812

[mjaa036-B2] Boehnke K. , Iversen P.W., Schumacher D., et al (2016). Assay establishment and validation of a high-throughput screening platform for three-dimensional patient-derived colon cancer organoid cultures. J. Biomol. Screen. 21, 931–941. 27233291 10.1177/1087057116650965 PMC5030729

[mjaa036-B3] Breslin S. , O'Driscoll L. (2013). Three-dimensional cell culture: the missing link in drug discovery. Drug Discov. Today 18, 240–249. 23073387 10.1016/j.drudis.2012.10.003

[mjaa036-B4] Broach J.R. , Thorner J. (1996). High-throughput screening for drug discovery. Nature 384, 14–16. 8895594

[mjaa036-B5] Canon J. , Rex K., Saiki A.Y., et al (2019). The clinical KRAS(G12C) inhibitor AMG 510 drives anti-tumour immunity. Nature 575, 217–223. 31666701 10.1038/s41586-019-1694-1

[mjaa036-B6] Carnero A. (2006). High throughput screening in drug discovery. Clin. Transl. Oncol. 8, 482–490. 16870538 10.1007/s12094-006-0048-2

[mjaa036-B7] Castellano E. , Sheridan C., Thin M.Z., et al (2013). Requirement for interaction of PI3-kinase p110α with RAS in lung tumor maintenance. Cancer Cell 24, 617–630. 24229709 10.1016/j.ccr.2013.09.012 PMC3826036

[mjaa036-B8] Castellon E.A. (2019). Patient-derived organoids: new co-clinical model to predict treatment response in cancer? Oral Dis. 25, 928–930. 30281877 10.1111/odi.12988

[mjaa036-B9] Chan L.L. , McCulley K.J., Kessel S.L. (2017). Assessment of cell viability with single-, dual-, and multi-staining methods using image cytometry. Methods Mol. Biol. 1601, 27–41. 28470515 10.1007/978-1-4939-6960-9_3

[mjaa036-B10] Clevers H. (2016). Modeling development and disease with organoids. Cell 165, 1586–1597. 27315476 10.1016/j.cell.2016.05.082

[mjaa036-B11] Crespo M. , Vilar E., Tsai S.Y., et al (2017). Colonic organoids derived from human induced pluripotent stem cells for modeling colorectal cancer and drug testing. Nat. Med. 23, 878–884. 28628110 10.1038/nm.4355 PMC6055224

[mjaa036-B12] Drilon A. , Schoenfeld A.J., Arbour K.C., et al (2019). Exceptional responders with invasive mucinous adenocarcinomas: a phase 2 trial of bortezomib in patients with KRAS G12D-mutant lung cancers. Cold Spring Harb. Mol. Case Stud. 5, a003665. 30936194 10.1101/mcs.a003665 PMC6549573

[mjaa036-B13] Drost J. , Clevers H. (2018). Organoids in cancer research. Nat. Rev. Cancer 18, 407–418. 29692415 10.1038/s41568-018-0007-6

[mjaa036-B14] Ferlay J. , Soerjomataram I., Dikshit R., et al (2015). Cancer incidence and mortality worldwide: sources, methods and major patterns in GLOBOCAN 2012. Int. J. Cancer 136, E359–E386. 25220842 10.1002/ijc.29210

[mjaa036-B15] Fox S. , Farr-Jones S., Yund M.A. (1999). High throughput screening for drug discovery: continually transitioning into new technology. J. Biomol. Screen. 4, 183–186. 10838437 10.1177/108705719900400405

[mjaa036-B16] Gao D. , Vela I., Sboner A., et al (2014). Organoid cultures derived from patients with advanced prostate cancer. Cell 159, 176–187. 25201530 10.1016/j.cell.2014.08.016 PMC4237931

[mjaa036-B17] Green S. , Trejo C.L., McMahon M. (2015). PIK3CA^H1047R^ accelerates and enhances KRAS^G12D^-driven lung tumorigenesis. Cancer Res. 75, 5378–5391. 26567140 10.1158/0008-5472.CAN-15-1249 PMC4681648

[mjaa036-B18] Griner L.M. , Gampa K., Do T., et al (2018). Generation of high-throughput three-dimensional tumor spheroids for drug screening. J. Vis. Exp. 10.3791/57476. PMC6235107 30247463

[mjaa036-B19] Hou S. , Tiriac H., Sridharan B.P., et al (2018). Advanced development of primary pancreatic organoid tumor models for high-throughput phenotypic drug screening. SLAS Discov. 23, 574–584. 29673279 10.1177/2472555218766842 PMC6013403

[mjaa036-B20] Huch M. , Bonfanti P., Boj S.F., et al (2013). Unlimited in vitro expansion of adult bi-potent pancreas progenitors through the Lgr5/R-spondin axis. EMBO J. 32, 2708–2721. 24045232 10.1038/emboj.2013.204 PMC3801438

[mjaa036-B21] Inglese J. , Johnson R.L., Simeonov A., et al (2007). High-throughput screening assays for the identification of chemical probes. Nat. Chem. Biol. 3, 466–479. 17637779 10.1038/nchembio.2007.17

[mjaa036-B22] Jiang L. , Tixeira R., Caruso S., et al (2016). Monitoring the progression of cell death and the disassembly of dying cells by flow cytometry. Nat. Protoc. 11, 655–663. 26938116 10.1038/nprot.2016.028

[mjaa036-B23] Johns M.A. , Meyerkord-Belton C.L., Du Y., et al (2014). The Emory Chemical Biology Discovery Center: leveraging academic innovation to advance novel targets through HTS and beyond. Comb. Chem. High Throughput Screen. 17, 290–296. 24409950 10.2174/1386207317666140109125415

[mjaa036-B24] Katano T. , Ootani A., Mizoshita T., et al (2013). Establishment of a long-term three-dimensional primary culture of mouse glandular stomach epithelial cells within the stem cell niche. Biochem. Biophys. Res. Commun. 432, 558–563. 23485463 10.1016/j.bbrc.2013.02.051

[mjaa036-B25] Kim M. , Mun H., Sung C.O., et al (2019). Patient-derived lung cancer organoids as in vitro cancer models for therapeutic screening. Nat. Commun. 10, 3991. 31488816 10.1038/s41467-019-11867-6 PMC6728380

[mjaa036-B26] Li L. , Knutsdottir H., Hui K., et al (2019). Human primary liver cancer organoids reveal intratumor and interpatient drug response heterogeneity. JCI Insight 4, e121490. 10.1172/jci.insight.121490 PMC6413833 30674722

[mjaa036-B27] Li X. , Nadauld L., Ootani A., et al (2014). Oncogenic transformation of diverse gastrointestinal tissues in primary organoid culture. Nat. Med. 20, 769–777. 24859528 10.1038/nm.3585 PMC4087144

[mjaa036-B28] Luo J. , Emanuele M.J., Li D., et al (2009). A genome-wide RNAi screen identifies multiple synthetic lethal interactions with the Ras oncogene. Cell 137, 835–848. 19490893 10.1016/j.cell.2009.05.006 PMC2768667

[mjaa036-B29] Madoux F. , Tanner A., Vessels M., et al (2017). A 1536-well 3D viability assay to assess the cytotoxic effect of drugs on spheroids. SLAS Discov. 22, 516–524. 28346088 10.1177/2472555216686308

[mjaa036-B30] Mo X. , Tang C., Niu Q., et al (2019). HTiP: high-throughput immunomodulator phenotypic screening platform to reveal IAP antagonists as anti-cancer immune enhancers. Cell Chem. Biol. 26, 331–339.e3. 30639259 10.1016/j.chembiol.2018.11.011 PMC6501824

[mjaa036-B31] Ootani A. , Li X., Sangiorgi E., et al (2009). Sustained in vitro intestinal epithelial culture within a Wnt-dependent stem cell niche. Nat. Med. 15, 701–706. 19398967 10.1038/nm.1951 PMC2919216

[mjaa036-B32] Sachs N. , de Ligt J., Kopper O., et al (2018). A living biobank of breast cancer organoids captures disease heterogeneity. Cell 172, 373–386.e10. 29224780 10.1016/j.cell.2017.11.010

[mjaa036-B33] Saito Y. , Muramatsu T., Kanai Y., et al (2019). Establishment of patient-derived organoids and drug screening for biliary tract carcinoma. Cell Rep. 27, 1265–1276.e4. 31018139 10.1016/j.celrep.2019.03.088

[mjaa036-B34] Schutte M. , Risch T., Abdavi-Azar N., et al (2017). Molecular dissection of colorectal cancer in pre-clinical models identifies biomarkers predicting sensitivity to EGFR inhibitors. Nat. Commun. 8, 14262. 28186126 10.1038/ncomms14262 PMC5309787

[mjaa036-B35] Skardal A. , Shupe T., Atala A. (2016). Organoid-on-a-chip and body-on-a-chip systems for drug screening and disease modeling. Drug Discov. Today 21, 1399–1411. 27422270 10.1016/j.drudis.2016.07.003 PMC9039871

[mjaa036-B36] Steckel M. , Molina-Arcas M., Weigelt B., et al (2012). Determination of synthetic lethal interactions in KRAS oncogene-dependent cancer cells reveals novel therapeutic targeting strategies. Cell Res. 22, 1227–1245. 22613949 10.1038/cr.2012.82 PMC3411175

[mjaa036-B37] Sumi T. , Hirai S., Yamaguchi M., et al (2018). Survivin knockdown induces senescence in TTF1-expressing, KRAS-mutant lung adenocarcinomas. Int. J. Oncol. 53, 33–46. 29658609 10.3892/ijo.2018.4365 PMC5958877

[mjaa036-B38] Suzuki K. , Murano T., Shimizu H., et al (2018). Single cell analysis of Crohn's disease patient-derived small intestinal organoids reveals disease activity-dependent modification of stem cell properties. J. Gastroenterol. 53, 1035–1047. 29374777 10.1007/s00535-018-1437-3 PMC6132922

[mjaa036-B39] van de Wetering M. , Francies H.E., Francis J.M., et al (2015). Prospective derivation of a living organoid biobank of colorectal cancer patients. Cell 161, 933–945. 25957691 10.1016/j.cell.2015.03.053 PMC6428276

[mjaa036-B40] Verissimo C.S. , Overmeer R.M., Ponsioen B., et al (2016). Targeting mutant RAS in patient-derived colorectal cancer organoids by combinatorial drug screening. eLife 5, e18489. 10.7554/eLife.18489 PMC5127645 27845624

[mjaa036-B41] Vlachogiannis G. , Hedayat S., Vatsiou A., et al (2018). Patient-derived organoids model treatment response of metastatic gastrointestinal cancers. Science 359, 920–926. 29472484 10.1126/science.aao2774 PMC6112415

[mjaa036-B42] Weaver V.M. , Lelievre S., Lakins J.N., et al (2002). β4 integrin-dependent formation of polarized three-dimensional architecture confers resistance to apoptosis in normal and malignant mammary epithelium. Cancer Cell 2, 205–216. 12242153 10.1016/s1535-6108(02)00125-3 PMC2788997

[mjaa036-B43] Weeber F. , Ooft S.N., Dijkstra K.K., et al (2017). Tumor organoids as a pre-clinical cancer model for drug discovery. Cell Chem. Biol. 24, 1092–1100. 28757181 10.1016/j.chembiol.2017.06.012

[mjaa036-B44] Wood L.D. , Parsons D.W., Jones S., et al (2007). The genomic landscapes of human breast and colorectal cancers. Science 318, 1108–1113. 17932254 10.1126/science.1145720

[mjaa036-B45] Yang H. , Sun L., Liu M., et al (2018). Patient-derived organoids: a promising model for personalized cancer treatment. Gastroenterol. Rep. 6, 243–245. 10.1093/gastro/goy040 PMC6225812 30430011

[mjaa036-B46] Yao Y. , Xu X., Yang L., et al (2020). Patient-derived organoids predict chemoradiation responses of locally advanced rectal cancer. Cell Stem Cell 26, 17–26.e6. 31761724 10.1016/j.stem.2019.10.010

[mjaa036-B47] Zhang J.H. , Chung T.D., Oldenburg K.R. (1999). A simple statistical parameter for use in evaluation and validation of high throughput screening assays. J. Biomol. Screen. 4, 67–73. 10838414 10.1177/108705719900400206

[mjaa036-B48] Zhu J.W. , Nagasawa H., Nagura F., et al (2000). Elucidation of strict structural requirements of brefeldin A as an inducer of differentiation and apoptosis. Bioorg. Med. Chem. 8, 455–463. 10722169 10.1016/s0968-0896(99)00297-7

